# Comparison of Methods to Correct Survival Estimates and Survival Regression Analysis on a Large HIV African Cohort

**DOI:** 10.1371/journal.pone.0031706

**Published:** 2012-02-23

**Authors:** Julie Henriques, Mar Pujades-Rodriguez, Megan McGuire, Elisabeth Szumilin, Jean Iwaz, Jean-François Etard, René Ecochard

**Affiliations:** 1 Service de Biostatistique, Hospices Civils de Lyon, Lyon, France; 2 Université de Lyon, Lyon, France; 3 Université Lyon 1, Villeurbanne, France; 4 Equipe Biostatistique-Santé, Centre National de la Recherche Scientifique-Unité Mixte de Recherche 5558 Laboratoire de Biométrie et Biologie Evolutive, Villeurbanne, France; 5 Epicentre, Paris, France; 6 Médecins Sans Frontières, Paris, France; 7 Institut de Recherche pour le Développement-Université de Montpellier Unité Mixte Internationale 233 TransVIHMI, Montpellier, France; Boston University, United States of America

## Abstract

**Objective:**

The evaluation of HIV treatment programs is generally based on an estimation of survival among patients receiving antiretroviral treatment (ART). In large HIV programs, loss to follow-up (LFU) rates remain high despite active patient tracing, which is likely to bias survival estimates and survival regression analyses.

**Methods:**

We compared uncorrected survival estimates derived from routine program data with estimates obtained by applying six correction methods that use updated outcome data by a field survey targeting LFU patients in a rural HIV program in Malawi. These methods were based on double-sampling and differed according to the weights given to survival estimates in LFU and non-LFU subpopulations. We then proposed a correction of the survival regression analysis.

**Results:**

Among 6,727 HIV-infected adults receiving ART, 9% were LFU after one year. The uncorrected survival estimates from routine data were 91% in women and 84% in men. According to increasing sophistication of the correction methods, the corrected survival estimates ranged from 89% to 85% in women and 82% to 77% in men. The estimates derived from uncorrected regression analyses were highly biased for initial tuberculosis mortality ratios (RR; 95% CI: 1.07; 0.76–1.50 vs. 2.06 to 2.28 with different correction weights), Kaposi sarcoma diagnosis (2.11; 1.61–2.76 vs. 2.64 to 3.9), and year of ART initiation (1.40; 1.17–1.66 vs. 1.29 to 1.34).

**Conclusions:**

In HIV programs with high LFU rates, the use of correction methods based on non-exhaustive double-sampling data are necessary to minimise the bias in survival estimates and survival regressions.

## Introduction

In the last five years, antiretroviral treatment (ART) programs have scaled-up in Sub-Saharan Africa to provide ART to millions of people. One of the main indicators used to evaluate the effectiveness of these programs is survival after ART initiation; however, this indicator is often biased because of underreporting and unrecorded deaths [1].

Several approaches have been proposed to minimise the attrition of study cohorts and ascertain the vital status of lost to follow-up (LFU) patients [2]–[5]. However, high rates of loss to follow-up represent a persistent challenge in program evaluation [6] and, there is yet no validated and commonly accepted method to analyse program data taking into account loss to follow-up data in the absence of vital registration systems.

Generally, program evaluations use estimates of the probability of remaining in care considering deaths and losses to follow-up as program failures [7]. These estimates may be difficult to interpret. Indeed, the death rate is frequently higher among LFU than among non-LFU patients for several reasons [8]–[10]: i) death can be the cause of the loss to follow-up; ii) patients prone to loss to follow-up might be frailer than the others and have higher risks of death; and, iii) after a few weeks or months without treatment, LFU patients become frailer than the others.

The traditional (uncorrected) approach considers that loss to follow-up is equivalent to administrative censoring at loss to follow-up date. Whenever human and financial resources are available, a double-sampling approach is used to ascertain the outcome of all or a subset of LFU patients and to correct the death rates either by updating the routine information or by applying a weighted average of the death rates observed among LFU and non-LFU patients [11]. In the absence of resources, external data from meta-analyses may be used to estimate the death rate among LFU patients [12]. More recent and sophisticated correction methods based on double-sampling have been shown optimal and provided unbiased estimations under some assumptions [13], [14]. Whenever no correction or a non-optimal correction is made, the magnitude of the bias may be significant.

Within the context of cohort attrition, some methods for regression analysis [15] are able to provide unbiased results when the probability of loss to follow-up does not change over the follow-up duration. An alternative method used herein provides also unbiased results even when the probability of loss to follow-up changes over time after follow-up onset.

In the present study, we use six different methods to correct data from a large HIV program in rural Malawi and compare the corresponding estimates of one-year survival to quantify bias reduction. We also propose a method to correct survival regression analyses.

## Methods

### The HIV program in Chiradzulu and data sources

Since 2001, Médecins Sans Frontières (MSF) and the Malawian Ministry of Health and Population have provided free ART to HIV-infected persons in Chiradzulu district, Malawi. Individual basic socio-demographic, clinico-immunological, and treatment data were collected at each clinical visit.

Later, a survey was conducted, as part of an internal audit of programme activities, to trace LFU patients and determine their outcomes and reasons for care discontinuation. All patients with an available address in Chiradzulu district and followed-up in one of MSF-supported health facilities were traced. Eleven district-wide catchment areas were defined according to the access to the facilities where care was provided. One survey worker from the community was hired within each catchment area and paid during the whole survey period. The survey workers had to cover the catchment areas by teams of two (on bicycles with car support when necessary) and each worker, in turn, was the team leader in his catchment area. All the workers were supervised by a long-term MSF worker. LFU patients were traced at least three times before the end of the ten-day search allotted for each catchment area. Various findings from this survey have been reported elsewhere [5].

In the present study, we analysed routine monitoring data on 6,727 ART-naive patients initiated on ART in the Chiradzulu program between July 2004 and July 2007 and aged 15 years or more at ART initiation. To facilitate comparisons of our results with previous literature, we limited the analysis to the first year after ART initiation. Thus, patient follow-up started at ART initiation and was censored at the earliest of the following dates: house moving, last clinical visit, or end of a one-year follow-up. A patient was considered LFU if he (she) missed a scheduled appointment by more than one month. The same censoring, one year after ART initiation, was used for traced patients.

**Figure 1:**
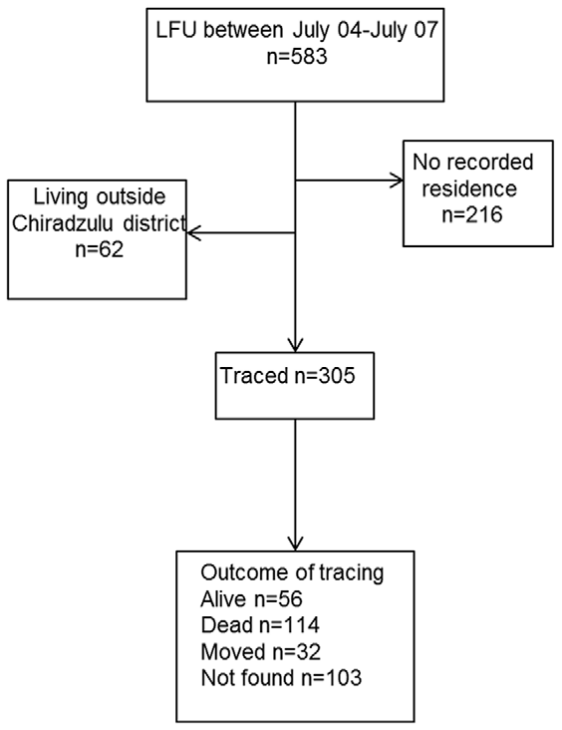
Flow chart of the study cohort.

During the study period, 610 (9.1%) patients died, 583 (8.7%) were LFU, and 413 (6.1%) were transferred out of the program. Among LFU patients, 305 (52%) could be traced and the vital status was ascertained for 202 (66%) (Figure 1).

### Statistical analyses

The characteristics of LFU and non-LFU patients were first described and compared using proportions and chi-square tests for categorical variables and medians, interquartile ranges (IQRs), and Kruskal-Wallis tests for continuous variables.

Patient survival estimates obtained using six different data correction methods were compared to the estimates obtained from uncorrected monitoring-program data. The six correction methods were: 1) the updated-dataset approach, 2) the stratified Kaplan-Meier approach, 3) the nomogram approach, 4) the time-dependent stratified Kaplan-Meier approach, 5) the time- and frailty-dependent stratified Kaplan-Meier approach, and, 6) a regression method corrected for missing information on death (Table 1). Survivals were estimated separately for men and women. Table 2 provides a list of assumptions necessary to obtain unbiased estimates.

**Table 1:** Methods to correct survival estimates.

Method		Presentation
1	Updated dataset	The information obtained on the subset of LFU patients who were traced and whose vital status was ascertained was used to update the dataset.
2	Stratified Kaplan-Meier	First, the death rates were calculated separately for non-LFU and for LFU patients who were traced and whose vital status was ascertained. These death rates were then combined to yield a weighted average. The weights used were the proportions of LFU and non-LFU patients at the end of the study.
3	Nomogram	See reference 12
4	Time-dependant stratified Kaplan-Meier	The death rates were first separately calculated for non-LFU and for LFU patients who were traced and whose vital status was ascertained at each event-time. These death rates were then combined to yield a weighted average. The weights used were the proportions of LFU and non-LFU patients at each event-time.
5	Time- and frailty-dependent stratified Kaplan-Meier	Step 1: A Cox proportional hazards model was used to identify factors predictive of LFU. The linear predictor of this model was calculated. Quintiles of this linear predictor were used to create strata of subject having the same propensity to be lost-to-follow-up. Step 2: Within each stratum, the death rates of non-LFU and traced LFU patients were computed. A global death rate was finally obtained combining all strata estimators.
6	Regression analysis	Step 1: A weighted logistic regression was applied to a dataset limited to dead patients using both the first-phase and the second-phase sample. This logistic regression predicted the probability of a death to be reported as a function of the covariates. This predicted probability was considered as a sensitivity. Step 2: The follow-up of each patient was split into successive time periods of one month each. Step 3: A Poisson regression model was used to estimate the death rate within each month period, the logarithm of the sensitivity being included in a standard Poisson regression model as an offset. Thus, the number of observed deaths was supposed to follow a Poisson distribution having as mean the product: number of patients at risk×rate of death×sensitivity. NB. The weight used in step 1 was 1 for patients whose death was identified in the first sample. The weight given to patients in the second-phase sample was used to take into account that only a subset of LFU patients were traced and had their vital status ascertained
	+wt1	Weight = 1; i.e., ignores that only a subset of LFU patients were traced and had their vital status ascertained.
	+wt2	Weight = the inverse of the proportion of patients traced and who had their vital status ascertained (see method 2)
	+wt3	Weight = the inverse of the proportion of patients traced and who had their vital status ascertained, at each time band (see method 4)

**Table 2:** Conditions to be fulfilled to obtain unbiased estimates.

		Condition			
Method		Patients traced and who had their vital status ascertained are representative of LFU patients	The death rate among LFU equals that of non-LFU	No change in the probability of being LFU over time	The probability of LFU does not depend on the covariates
1	Updated dataset	✓	✓	✓	✓
2	Stratified Kaplan-Meier	✓		✓	✓
3	Nomogram	✓		✓	✓
4	Time-dependent stratified Kaplan-Meier	✓			✓
5	Time and frailty dependent stratified Kaplan-Meier	✓			
6	Regression analysis				
	+wt1	✓	✓	✓	✓
	+wt2	✓		✓	✓
	+wt3	✓			✓

For the updated-dataset approach, the data on outcome were updated using the data collected during the survey ignoring that a subset of LFU patients cannot be traced or found. Moreover, a sensitivity analysis was carried to investigate the way in which the results would be modified if all LFU patients were assumed to die immediately after the last visit.

For the stratified Kaplan-Meier approach, mortality was first calculated separately for non-LFU patients and LFU patients who were traced and had their vital status ascertained. The two mortalities were then combined applying weights that correspond to the proportions LFU and non-LFU patients recorded at one year after ART initiation.

With the nomogram approach, graphical estimates of the death rates were obtained taking into account mortality in LFU and non-LFU patients up to time *t* [12] and the proportion of LFU patients at end of follow-up.

The time-dependent stratified Kaplan-Meier approach was used to account for changes in the probability of loss of follow-up over time [13]. The estimated death rate at each time point was calculated using the updated weighted averages of the death rates among non-LFU patients and among LFU patients who were traced and had their vital status ascertained. The weights were, respectively, the proportions of non-LFU and LFU patients at each time point *t*. This method ignores a crucial heterogeneity between the patients at time *t*; actually, LFU patients could be frailer than regularly followed-up ones.

The time- and frailty-dependent stratified Kaplan-Meier approach was proposed by An et al. [14] to account for that frailty. It was applied using weighted averages within strata of subjects having the same propensity to be lost-to-follow-up.

Regression methods were finally used to compensate for the lack of information on death. In a first step, a logistic regression was applied to a dataset that included only deceased patients. In this logistic regression, the dependent variable was coded 1 when death was ascertained from the routine program data and 0 when death was ascertained after tracing LFU patients. This logistic regression provided an estimate of the probability for a death to be recorded in routine program data for each subgroup of patients. This probability may be considered as the sensitivity of the routine program to detect death; i.e., this sensitivity corresponds to the likelihood of a death to be recorded in routine program data. In the model, we included sex, age (as a continuous variable), tuberculosis and Kaposi sarcoma diagnosis, CD4 cell count at ART initiation (≤ or >150 cells/mm^3^), the year of therapy start (2004–2005 or 2006–2007), and the delay between ART initiation and death. Note that the national ART program was implemented in 2004 in centralized hospitals throughout Malawi (approximately 55 sites). By the end of 2007, 109 sites were providing care. The Chiradzulu program scaled up the decentralization of HIV services in 2006. This was the reason for the choice of 2006 as a threshold.

Three weighting options were applied to the logistic regression to take into account that only a subgroup of LFU patients were traced: 1) weight set to one (wt1), ignoring that only a subset of LFU patients were traced and had their vital status ascertained; 2) weight set to the inverse of the proportion of the above-mentioned subset among LFU patients (wt2); and 3) weight set to the inverse of the time-specific proportion of the above-mentioned subset among LFU patients (wt3), allowing for changes in this proportion over time. Poisson regression models were then fitted after splitting individual patient follow-ups into successive one-month time periods. Observed deaths in routine program data were assumed to follow a Poisson distribution whose mean was equal to the product of three factors: number of patients at risk×rate of death×sensitivity. The results are presented as adjusted mortality ratios (RR) with 95% confidence intervals (CI).

All the analyses were conducted using Stata 10 (StataCorp LP, College Station, TX, USA), except the time- and frailty-dependent stratified Kaplan-Meier approach for which a specific program, developed in R software, was provided by the author of the original publication [14].

### Ethics

The protocols of the Chiradzulu project were approved within the framework of formal agreements between MSF and the Malawian Ministry of Health. The present observational study was conducted under the supervision of the Malawi National Health Science Research Committee with an agreement on collection and use of routine programmatic data for monitoring and evaluation. The study type did not require a formal submission for ethical approval.

## Results

### Patient characteristics at ART initiation

From July 2004 to July 2007, 6,727 patients aged 15 years or older were initiated on ART in the Chiradzulu program. At initiation, the median patient age was 35.2 years (IQR: 30.0–43.0), 65% of the patients were women, 5% had tuberculosis, and 4.7% had Kaposi sarcoma (Table 3). Among the 3,271 patients with available CD4 cell count at ART initiation, 24.9% had less than 150 cells/mm^3^.

**Table 3:** Patient characteristics and mortality among lost to follow-up and non-lost-to-follow-up patients.

Characteristics	Cohort	Non-LFU	LFU	p-value *
Number	6,727	6,144	583	
Women	4,372 (65.0%)	4,055 (66.0%)	317 (54.4%)	0.0001
Age at ART start	35.2 [30.0;43.0]	35.2 [30.0;43.1]	34.3 [29.0;42.2]	0.0138
Tuberculosis at ART start	349 (5.2%)	302 (4.9%)	47 (8.1%)	0.001
Kaposi sarcoma at ART start	315 (4.7%)	244 (4.0%)	71 (12.2%)	0.0001
Year of ART initiation				0.0001
2004–2005	2,455 (36.5%)	2,170 (35.3%)	285 (48.9%)	
2006–2007	4,272 (63.5%)	3,974 (64.7%)	298 (51.1%)	
First CD4 cell count				0.0001
<150 cells/mm^3^	1,673 (24.9%)	1,573 (25.6%)	100 (17.1%)	
≥150 cells/mm^3^	1,598 (23.7%)	1,530 (24.9%)	68 (11.7%)	
Missing	3,456 (51.4%)	3,041 (49.5%)	415 (71.2%)	
Patients with known vital status	6,346	6,144	202	
Deaths among patients with known vital status in the first year of ART	731 (10.9%)	610 (9.9%)	120 (20.6%)	0.0001

All values are expressed as number (percentage) but “Age at ART initiation” expressed as Median [Interquartile range].

*Pearson chi-square test was used for binary covariates and Student t-test for continuous covariates.

The proportion of men was higher among LFU than among non-LFU patients (66% vs. 54.4%; p-value = 0.0001). Compared to patients in care, LFU patients were younger (median age 34.3 vs. 35.2, p-value = 0.0138) and more frequently diagnosed with tuberculosis (8.1% vs. 4.9%; p-value = 0.0001) or Kaposi sarcoma (12.2% vs. 4%; p-value = 0.0001) at ART start.

Sixty percent of the 202 LFU patients who were traced and had their vital status ascertained during the survey were reported dead whereas just 10% of non-LFU patients died during the first year of ART (p-value = 0.0001).

Within the context of these comparisons, it should be noted that the differences between LFU and non-LFU patients should probably not be evaluated with p-values because these differences are due to selection not to sampling error.

### Comparison between survival estimates with and without correction

**Figure 2:**
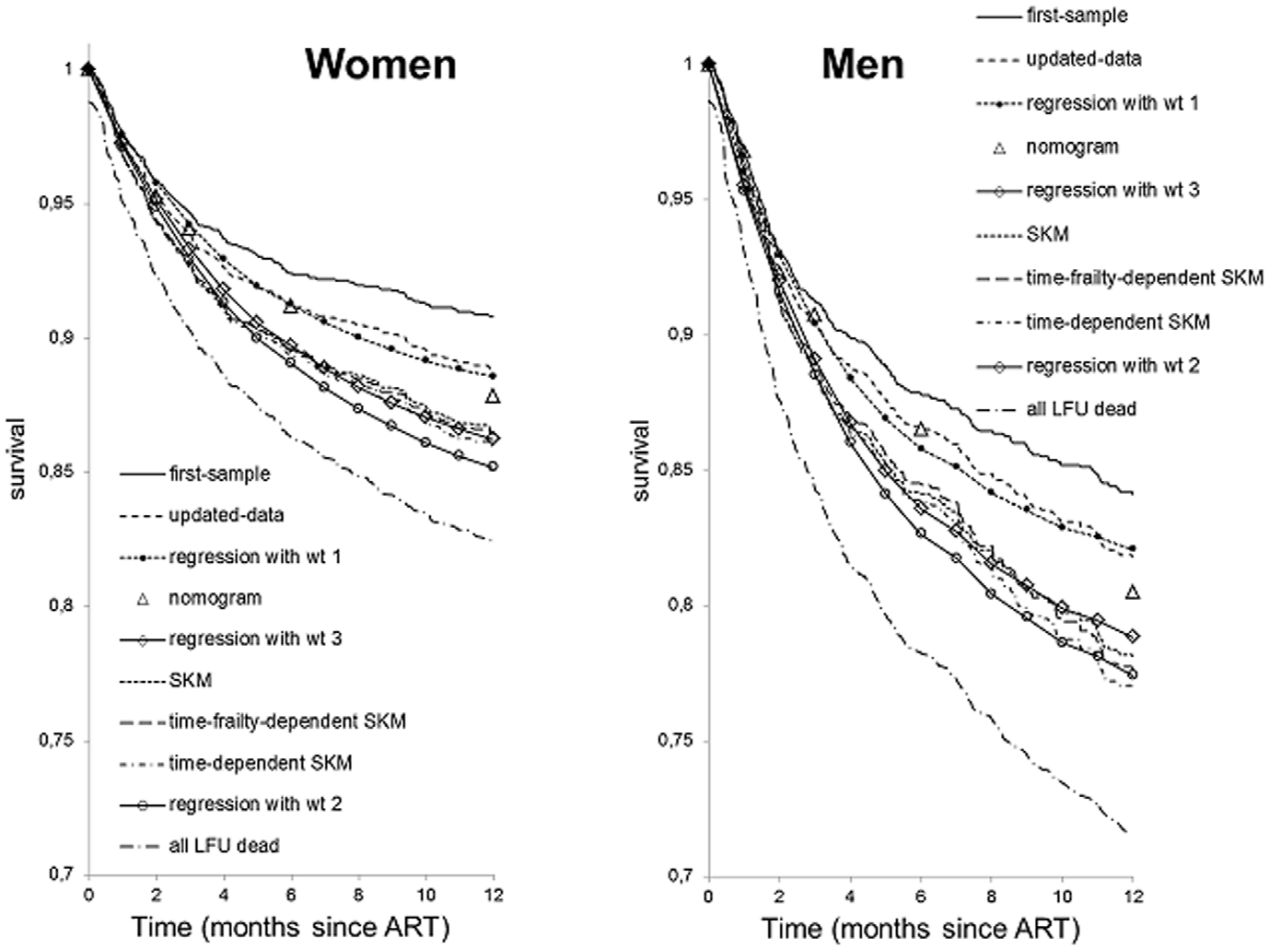
Patient survival in men and women. The upper and lower packs of line identifiers correspond respectively to the visibly grouped graphs or methods. SKM = stratified Kaplan-Meier method, wt = weight.

As expected, with the uncorrected method based on the use of routine program data, the highest one-year survival estimates were 84% in men and 91% in women (Figure 2).

With the updated-dataset method and the regression method with wt1 (i.e., ignoring that only a subset of LFU patients were traced and had their vital status ascertained), the corrected one-year estimates were 2% lower in men and women. The estimates obtained with the time-dependent stratified Kaplan-Meier approach as with the survival regression were 7% and 6% lower than the uncorrected estimates in men and women, respectively. With the nomogram method, the results were intermediate; i.e., the corrected estimates were about 3% and 2.5% lower than the uncorrected ones in men and women, respectively.

With the updated-dataset method, supposing that, for the sensitivity analysis, all LFU patients died immediately after their last visit, the one-year survival estimates were 72% in men and 82% in women. These estimates were lower than the estimates obtained using alternative methods.

### Comparisons between regression methods with and without correction

Table 4 shows the results of the regressions of the death rate on the studied factors, with and without correction for lack of information on death.

**Table 4:** Relative mortality estimated with Poisson regression on uncorrected or corrected data.

Characteristics at ART initiation	Uncorrected	Weight 1	Weight 2	Weight 3
Duration of follow-up (months)	0.80 [0.77–0.82]	0.85 [0.82–0.87]	0.87 [0.84–0.90]	0.86 [0.93–0.89]
Sex (women vs. men)	0.62 [0.53–0.73]	0.67 [0.57–0.79]	0.69 [0.58–0.81]	0.67 [0.57–0.79]
CD4 cell count (vs. <150 cells/mm^3^)				
≥150 cells/mm^3^	0.64 [0.48–0.86]	0.69 [0.51–0.92]	0.67 [0.51–0.90]	0.66 [0.49–0.87]
Missing	1.51 [1.23–1.86]	1.33 [1.07–1.64]	1.41 [1.14–1.74]	1.43 [1.16–1.77]
Tuberculosis (Yes vs. no)	1.07 [0.76–1.50]	2.28 [1.62–3.22]	2.27 [1.61–3.21]	2.06 [1.46–2.90]
Kaposi sarcoma (Yes vs. no)	2.11 [1.61–2.76]	2.96 [2.25–3.89]	3.09 [2.35–4.06]	2.64 [2.01–3.48]
Year of ART start (2006–07 vs. 2004–05)	1.40 [1.17–1.66]	1.34 [1.12–1.60]	1.33 [1.11–1.59]	1.29 [1.08–1.54]

Three different weightings were considered for correction: Weight 1 = 1, Weight 2 = the inverse of the proportion of LFU patients who were traced and had their vital status ascertained, Weight 3 = the inverse of the time-specific proportion of them.

All the values are expressed as mortality ratios [95% confidence interval].

Uncorrected mortality ratios decreased with time of follow-up (uncorrected RR = 0.80, 95% CI; 0.77–0.88 per month of follow-up). These ratios were lower in women vs. men (0.62; 0.53–0.73) and lower in patients with high vs. low CD4 cell counts (0.64; 0.48–0.86 for >150 vs. ≤150 cells/mm^3^). These ratios were also higher in patients with vs. without an initial diagnosis of Kaposi sarcoma (2.11; 1.61–2.76) and in those who started ART more recently (1.40; 1.17–1.66 for start in 2006–07 vs. 2004–05).

Compared to the uncorrected estimates, the estimates obtained with correction showed slower decreases in mortality over time (RR ranged from 0.85, 95% CI; 0.82–0.87 with wt1 to 0.87; 0.84–0.90 with wt2 regression) and lower mortality ratios in patients who started ART more recently (RR ranged from 1.29; 1.08–1.54 with wt3 to 1.34; 1.12–1.60 with wt1 regression, for 2006–07 vs. 2004–05). Higher ratios were observed in patients with vs. without initial diagnosis of Kaposi sarcoma (RR ranged from 2.64; 2.01–3.48 with wt3 to 3.09; 2.35–4.06 with wt2 regression) and in patients diagnosed with vs. without tuberculosis at ART start (RR ranged from 2.06; 1.46–2.90 with wt3 to 2.28; 1.62–3.22 with wt1 regression). In contrast, mortality ratios in patients with high vs. low CD4 cell counts were similar before and after correction (RR ranged from 0.66; 0.49–0.87 with wt3 to 0.69; 0.51–0.92 with wt1 regression, for >150 vs. ≤150 cells/mm^3^).

## Discussion

A number of previous studies have raised concerns regarding poor patient retention in HIV care and warned that ART program effectiveness could thus be overstated [16], [17]. Our findings illustrate the ways in which different statistical methods based on double-sampling can be used to correct survival estimates and how the reduction of bias varies according to the method used. In this large HIV treatment cohort in rural Malawi where survival estimates were 91% in women and 84% in men, the corrected estimates ranged from 89% and 82%, respectively, with the simplest methods, to 85% and 77%, respectively with the most sophisticated ones. These findings highlight the importance of accounting for the unknown mortality in LFU patients and the benefit of using sophisticated methods [14].

Broadly speaking, there are two ways of obtaining survival estimates. The first is “retrospective”; it uses outreach data –not collected for weighting– through double sampling but adjusts patient retention to be as representative as possible. The second is “prospective”; it identifies the sampling frame ahead of time, which ensures the representativeness of the double-sampled data by design. Each way has its proper use, but two advantages of the prospective approach are that it requires fewer assumptions and subject only to sampling error.

As in previous studies, we observed a higher risk of death among LFU patients than among patients retained in care [18]. The use of uncorrected survival estimates would have therefore led to an overestimation of the program performance. As described in a South African study, linking patient data from HIV programs to vital registers would be the ideal way to obtain unbiased estimates of patient survival [19], [20]. However, in many Sub-Saharan Africa settings, vital registration is not available, which requires the use of alternative correction methods. In the Chiradzulu program, the use of data from a survey conducted to ascertain the vital status of LFU patients and update the routine information did not succeed in correcting for loss of follow-up because of the small proportion of LFU patients who were traced and had their vital status ascertained.

The survival estimates given by the updated-dataset and the regression methods corrected for lack of information on death using the first weight option were only slightly lower that those obtained without correction. This is because these two methods ignore that a subset of LFU patients were not traced or their vital status not ascertained. Higher estimates were obtained using the nomogram and the stratified Kaplan-Meier approaches by applying to all LFU patients the estimated death rate observed among LFU patients who were traced and had their vital status ascertained. Two advantages of the nomogram are that it is easy to implement and does not require prior double-sampling because it uses external data. Nomogram-derived estimates can be considered as a preliminary correction; however, caution is necessary in the absence of double-sampling data.

The methods that take into account the proportion of LFU patients at each time point during follow-up gave lower survival estimates than the above-cited ones and may be considered as gold-standards [13].

The regression method with the weighting option 2 (wt2) proposed in the present study is close to another method proposed recently within the same context [15]. The regression method with the weighting option 3 (wt3) is a variant that accounts for changes in the probability of loss to follow-up over time [13]. In our cohort, the regression method proposed to correct survival estimates taking into account the uneven likelihood of outcome ascertainment among patients gave similar results to the uncorrected regression methods but only for some factors. The uncorrected survival regression analysis was sufficient to estimate the effects of factors such as the initial CD4 cell count; this highlights the importance of this predictor of mortality independently of patient gender or other factors. However, the mortality ratios related to tuberculosis doubled and the ratios related to Kaposi sarcoma diagnosis at ART start increased by 25–40% when corrected regression methods were used. These higher ratios may be explained by the expected increased risk of loss to follow-up and death among the patients presenting severe tuberculosis and Kaposi sarcoma disease. This finding highlights the need to correct survival regression models for the missing information on death within the context of program evaluation in large HIV cohorts with high loss to follow-up rates. However, this approach might be too complicated to be implemented in routine programs. Simpler statistical methods, such as the simulation-extrapolation method (SIMEX) [21], [22], could be good alternatives but they require double-sampling.

Survival regression assumed that the sensitivity to correctly identify deaths in the program was known and equal to the observed estimation; however, this assumption may be wrong. Structural equation modelling would avoid the need for separate estimations of sensitivity and the death rate and provide accurate confidence intervals.

The present study has several limitations. In our cohort, the proportion of patients who were traced and had their vital status ascertained represented only one third of all LFU patients. A study by Anglaret et al. suggested that unfound LFU patients are at higher risk of death than found ones [3]. This means that the approaches that assume that traced LFU patients are a representative sample of all LFU patients would overestimate survival. A selection process could have affected the distribution of the outcomes in the traced sample. Patients with no recorded residence could be more vulnerable to mortality or have poor access to health services. Patients living outside Chiradzulu district may also differ from the others; however, there was no evidence to suggest that a geographical selection would bias the results. Finally and interestingly, one out of six patients traced was not found. A number of recorded addresses were probably not correct but no address checking was carried out neither at inclusion nor when patients moved out of the district. It should also be noted that Chiradzulu district was one of the first places in Malawi to provide ART. As the access to treatment increased, a number of patients may have moved out of the district to receive care closer to their homes or home villages.

Some relevant patient characteristics, such as the WHO stage or the adherence to treatment, could not be used in the regression models because of missing or unavailable information. For example, the CD4 cell counts were not available in half of the patients. The lack of information on these counts is known to be frequent among patients with advanced HIV disease at ART start. This is why a category for missing CD4 count was included in the regression analysis even though this practice is not generally advisable. Finally, a comparison between long-term survival estimates (one year after ART start) obtained with various correction methods should be performed because the proportion of deaths among LFU patients and the factors associated with outcome ascertainment in a program are likely to change with the increase of time on ART.

In conclusion, evaluations of HIV programs with high loss to follow-up rates should be based on corrected survival estimates. Though all the correction methods proposed in this article may be carried out using standard statistical software, their routine use in field surveys is not straightforward. The future development of a simple application should help program managers use these methods in routine program evaluation.
